# Influence of cervical preflaring and root canal preparation on the fracture resistance of endodontically treated teeth

**DOI:** 10.1186/s12903-020-1050-8

**Published:** 2020-04-16

**Authors:** Paula Barcellos da Silva, Simone Ferreti Duarte, Murilo Priori Alcalde, Marco Antonio Húngaro Duarte, Rodrigo Ricci Vivan, Ricardo Abreu da Rosa, Marcus Vinícius Reis Só, Angela Longo do Nascimento

**Affiliations:** 1 Departamento de Odontologia Conservadora, Universidade Federal do Rio Grande do Sul, 2492 Ramiro Barcelos Street, Porto Alegre, RS 90035-003 Brazil; 2 Department of Endodontics, University of São Paulo, Bauru, SP Brazil

**Keywords:** Endodontics, Root canal preparation, X-ray microtomography

## Abstract

**Background:**

Evaluate the fracture resistance of endodontically treated teeth after cervical preflaring and root canal preparation and to assess the volume of the root canal and the amount of remaining root dentin before and after cervical preflaring.

**Methods:**

Forty-four mandibular incisors were selected using micro-CT scanning and distributed into 4 groups (*n* = 11) according to the instrument used for cervical preflaring: control group - no cervical preflaring; Gates Glidden – burs size #2 and #3; WXN – 25.07 Navigator instrument; and Easy – 25.08 ProDesign S instrument. Coronal opening was performed, and the canals were prepared with Wave One Gold Primary and filled with an epoxy-resin based sealer and gutta-percha cones. Micro-CT scans were performed before and after root canal instrumentation. All images were reconstructed and assessed for the thickness of mesial and distal root dentin at 3 mm and 5 mm from the cement -enamel junction and for the volume of cervical portion of the canal after preparation. Fracture resistance test was performed applying compressive loads at a crosshead speed of 0.5 mm/min, applied on the palatal aspect of specimens at 135° along the long axis of the tooth. The data were analyzed using ANOVA and Tukey’s test (*P* = .05).

**Results:**

Cervical preflaring and canal preparation reduced the dentin thickness (*P* < .05) and increased the canal volume (*P* < .05) in all groups at 3 mm an 5 mm. Cervical preflaring with Gates Gliden burs reduced the fracture resistance of endodontically treated teeth (*P* < .05).

**Conclusions:**

All instruments reduced the dentin thickness and increased the canal volume in the cervical at 3 mm and 5 mm. Gates Glidden reduced fracture resistance of mandibular incisors submitted to cervical preflaring, whereas NiTi instruments did not.

**Clinical relevance:**

Cervical preflaring assumes particular importance previously to the root canal preparation because it minimizes the occurrence of operative accidents, and permits more accurate determination of working length and the apical diameter.

## Background

All the current advances in Endodontics aimed to improve the quality of the root canal treatment and the clinical success rates. Nickel-titanium rotary and reciprocating instruments enabled better canal shaping, especially in curve and narrow root canals, because their design and flexibility [1].

Independently of the rotary or reciprocating system used for root canal preparation, cervical preflaring assumes particular importance because it minimizes the occurrence of operative accidents [2], reduces apical extrusion of debris [3], removes dentin interferences and allows free and straight access to the instrument along the working length [4]. For consequence, the risk of fracture of the file and apical deviations are minimized [5, 6]. Furthermore, after cervical preflaring, the working length and the apical diameter can be established more accurately [7, 8].

The main reasons to extract a tooth are related to periodontal disease, failures in endodontic treatment and vertical root fracture [9]. Root fractures can be originated after dental trauma or endodontic procedures such as chemomechanical preparation, filling techniques or retreatment procedures [10]. Some studies have assessed the influence of the thickness of dentinal walls after cervical preflaring and established that canal diameter at the cervical third must not exceed one third of the root width [5].

Several instruments are available in the market to perform the cervical preflaring. Traditionally, Gates Glidden, Largo, and LA Axxess burs have been employed for this purpose. A recent study associated Gates Gliden burs to higher incidence of dentin cracks in comparison with some NiTi systems used for cervical preparation [10]. Thus, some authors do not recommend cervical preflaring with these burs, once several NiTi instruments have been used in a crown-down approach [3].

Therefore, the aims of this study were: (1) to evaluate the fracture resistance of endodontically treated teeth submitted to cervical preflaring using different instruments and (2) to assess the volume of the root canal and the amount of remaining root dentin in the 3 mm and 5 mm below the enamel-cement junction after cervical preflaring and canal preparation. The null hypothesis was that would be no difference regarding the fracture resistance, volume of the root canals and amount of remaining dentin among the groups.

## Methods

### Sample selection and ethical aspects

Forty-four human mandibular incisors were selected and stored in distilled water at room temperature until their use in this study. The selected teeth were donated by patients for the research and the donation term were signed. Digital radiographs were performed to evaluate if all teeth met the inclusion criteria. Teeth with complete root formation, absence of root curvature or dentin cracks/fractures, only one canal, no previous endodontic treatment or root resorption were included. The excess moisture was removed and the specimens were scanned at an isotropic resolution of 11.88 lm using a high-resolution microtomography scanner with aluminium and copper filters (SkyScan 1172; Bruker-microCT, Bruker, Aartselaar, Belgium) at 100 kV and 100 lA. A baseline micro-CT scan was performed using a according to the parameters used by Barreto [11].

This study was submitted and approved by Research Ethics Committee of Federal University of Rio Grande do Sul, Porto Alegre, Brazil (#68322017.8.0000.5347).

### Root canal preparation and filling

Coronal opening was performed as usual using #1014 diamond burs (Fava Metalúrgica, São Paulo, Brazil) under water cooling. Next, canal scouting were performed with a size 10 and 15 K-file (Dentsply Maillefer, Ballaigues, Switzerland). Working length (WL) was established using a #15 K-flei (Dentsply Maillefer) wich was introduced into the canal until it became visible at the apical foramen and then subtracting 1 mm from this measurement.

All teeth were randomly divided into four experimental groups (*n* = 11) according to the instrument used for cervical preflaring and one control group: Control group - canals were prepared but without cervical preflaring; Gates Glidden group – burs size 2 and 3 (Dentsply Maillefer); WXN group – 25.07 instrument (Navigator; MEDIN, Nové Město na Moravě, Czech Republic); and Easy group – 25.08 instrument (ProDesign S; Easy Instrumentos Odontológicos, Belo Horizonte, Brazil).

Cervical preflaring in the Gates Glidden group was performed on a low-speed handpiece operating at 12.000 rpm, and a straight up-and-down motion was used up to 5 mm from the canal opening. Cervical preflaring in WXN and Easy groups were performed on an electric device operated at 350 rpm and torque of 2 N.cm. These instruments were used in an in-and-out motion.

The root canal preparation was performed with Wave One Gold Primary instruments (25.07) (Dentsply Sirona, York, EUA) in a reciprocating motion, using the speed and torque recommended by the manufacturer. Each instrument was used in five teeth. During canal preparation, the canals were irrigated with 5 mL of 2.5% of sodium hypochlorite (NaOCl) (Biodinâmica, Ibiporã, PR, Brazil) carried out with a syringe and 30-gauge needle (NaviTip, Ultradent, South Jordan, UT, USA) taken 1 mm short of the WL.

After instrumentation, the root canals were irrigated with 5 mL of 17% EDTA (Biodinâmica) for 3 min and rinsed with 2 mL distilled water and dried with absorbent sterile paper points. Root canal filling was performed using Tagger’s Hybrid technique. The Wave One Primary gutta-percha cones (Dentsply Sirona) were inserted into the canals to verify if they reach the WL and if they fit at the apical third. Subsequently, an epoxy-resin based sealer (Sealer Plus; MK Life, Porto Alegre, RS, Brazil) was mixed according to the manufacturer’s instructions and placed in the canal using a lentulo spiral. Finally, the gutta-percha cone was slowly inserted into the canal until it reached WL. Next, three FM gutta-percha cones were inserted passively. Finally, a McSpadden compactor #50 (Dentsply Maillefer) was coupled to a low-speed contra-angle and introduced passively into the root canal 4 mm short of the WL with forward-backward movements.

The excess of gutta-percha was removed with a heated plugger, and the coronal opening was cleaned with ethanol (Biodinâmica). Next, the coronal opening was etched with 37% phosphoric acid for 30 s, rinsed with 2 mL of distilled water for 30 s and restored using adhesive system (Scotchbond; 3 M ESPE, Saint Paul, MN, USA) and nanoparticulated composite resin (Filtek™ Z-350; 3 M ESPE). The restorative procedures were performed using the incremental filling technique. Each increment was light-cured for 40 s using a LED light source (Radii-cal, SDI, Bayswater, VC, Australia) with a power of 1200 mW/cm^2^.

All instrumentation and root canal filling procedures were performed by one single experienced operator and trained to use the instruments.

### Analysis of root canal enlargement and dentin thickness

A new micro-CT scan was performed as described before and the reconstructed images obtained pre and post-preparation were geometrically co-registered with the preoperative data sets using the DataViewer software v.1.5.2 (Bruker-microCT), allowing quantitative comparison of the morphological parameter before and after preparation. For this step, the CTan v.1.12 software was used to evaluate the root canal volume and the dentin thickness pre and post root canal preparation in the cervical portion. The dentin thickness (mm) was evaluated at 3 mm and 5 mm from the cement-enamel junction and the canal volume (mm^3^) only in the first 5 mm.

### Fracture resistance test

After restoration, the simulation of periodontal ligament was performed by teeth immersing in melted wax (Horus; Herpo ProdutosDentários, Petrópolis, RJ, Brazil) up to 1 mm below to the cementoenamel junction. After cooling, a 0.2 mm + 01 mm thick wax layer was obtained by coating the roots. Next, the specimens from all of the groups were embedded in plastic cylinders (16.5 mm inner diameter X 20 mm high) filled with a chemically cured acrylic resin (Dencrilay, Dencril, SP, Brazil) using the following steps: a) the specimen was fixed on a parallelometer, with the long axes of the teeth and cylinder parallel to each other and perpendicular to the ground, and b) the acrylic resin was prepared and poured inside the cylinder up to 1 mm below the cementoenamel junction.

After resin polymerization, the wax was removed from the root surface and the resin cylinder ‘sockets’ by using warm water for 2 s. The resin cylinders were filled with a polyether impression material (ImpregumTM Soft, 3 M ESPE) using a molding syringe. The teeth were re-inserted into their respective cylinder ‘sockets’, and any excess impression material was removed with a number 12 scalpel blade.

Fracture resistance tests were performed 48 h after removal of impression material. During this period, the specimens were kept in distilled water at 37 °C. Compressive loads were applied using a universal testing machine (EMIC DL 2000/700; São José dos Pinhais, PR, Brazil) at a crosshead speed of 0.5 mm/min, applied on the palatal aspect of specimens at 135° along the long axis of the tooth.

### Statistical analysis

The normality of the data was assessed by Shapiro Wilk Test. The two-dimensional and three-dimensional parameters were compared between and within groups using ANOVA and Tukey’s test (*P* < .05). The fracture resistance values were subjected to 1-way ANOVA and Tukey post-hoc tests. Statistical analysis was performed at a significance level of 5%.

## Results

Table 1 shows the means and standard deviations of dentin thickness, volume of root canal (mm^3^) of the first 5 mm below the cement-enamel and fracture resistance.

**Fig. 1:**
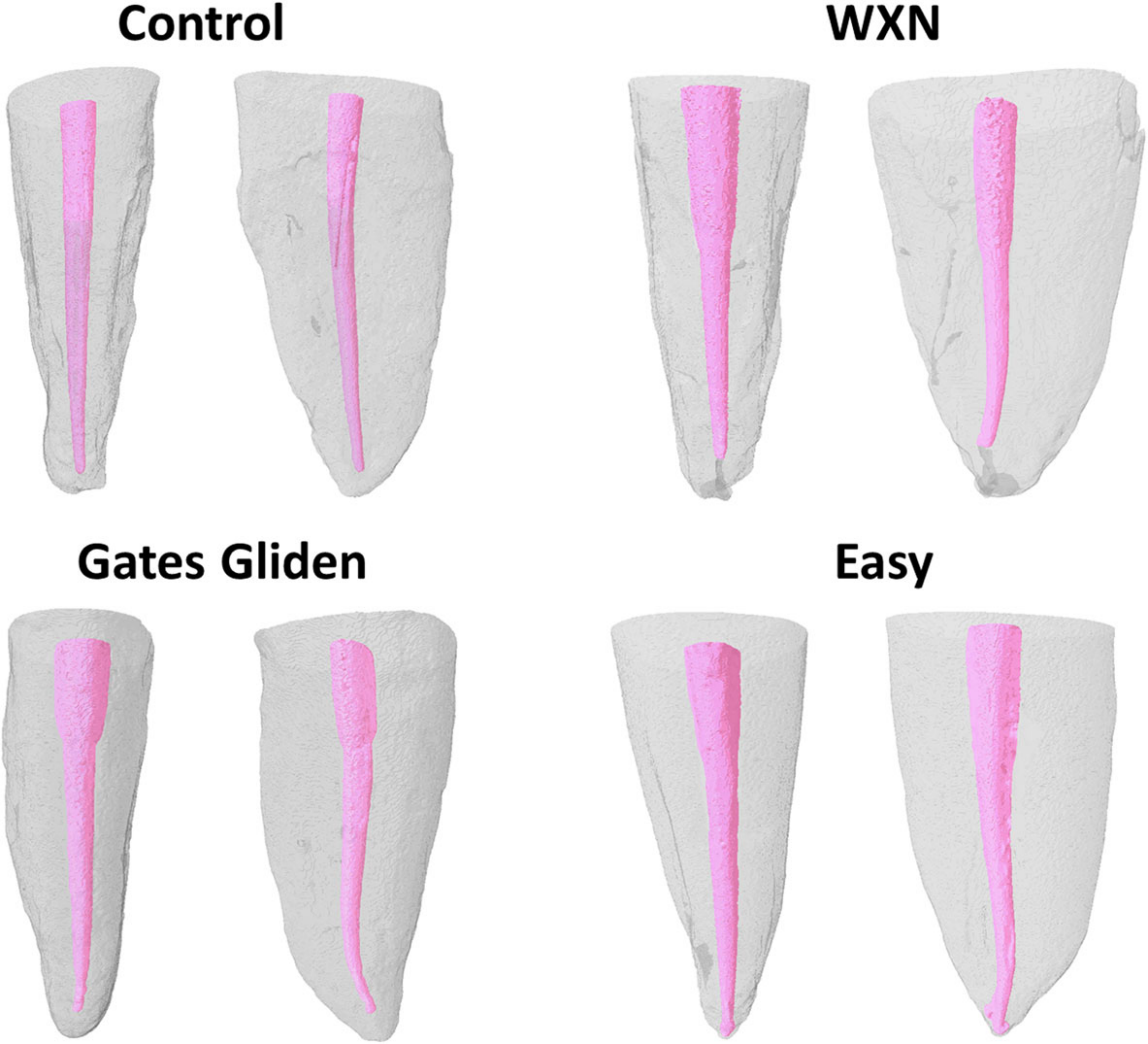
Micro-CT scans after cervical preflaring and root canal preparation. Note the hourglass format of the canal in the cervical third of Gates Gliden group

**Table 1:** Mean and standard deviation of dentin thickness (mm) and volume of root canal (mm^3^) in each group at 3 mm and 5 mm below the cement-enamel junction before and after root canal preparation and fracture resistance (N)

	3 mm Distal		3 mm Mesial		5 mm Distal		5 mm Mesial		Volume		Fracture resistance
	Before	After	Before	After	Before	After	Before	After	Before	After	
Control	1.39 ^a^ (±0.15)	1.09 ^b^ (±0.17)	1.32 ^a^ (±0.18)	1.12 ^b^ (±0.14)	1.32 ^a^ (±0.14)	1.06 ^b^ (±0.16)	1.32 ^a^ (±0.16)	1.07 ^b^ (±0.13)	1.37 ^a^ (±1.11)	3.11 ^b^ (±1.08)	581.12 ^A^ (±117.13)
Gates	1.39 ^a^ (±0.22)	1.17 ^b^ (±0.18)	1.36 ^a^ (±0.17)	1.16 ^b^ (±0.22)	1.24 ^a^ (±0.19)	1.06 ^b^ (±0.21)	1.21 ^a^ (±0.14)	1.02 ^b^ (±0.14)	1.34 ^a^ (±0.41)	2.61 ^b^ (±0.56)	404.59 ^B^ (±54.46)
WXN	1.45 ^a^ (±0.21)	1.24 ^b^ (±0.29)	1.51 ^a^ (±0.25)	1.25 ^b^ (±0.31)	1.31 ^a^ (±0.15)	1.11 ^b^ (±0.18)	1.37 ^a^ (±0.22)	1.15 ^b^ (±0.27)	1.90 ^a^ (±0.70)	3.16 ^b^ (±0.94)	551.47 ^A^ (±112.83)
Easy	1.57 ^a^ (±0.15)	1.23 ^b^ (±0.17)	1.46 ^a^ (±0.13)	1.09 ^b^ (±0.12)	1.39 ^a^ (±0.18)	1.07 ^b^ (±0.13)	1.39 ^a^ (±0.22)	1.03 ^b^ (±0.15)	1.65 ^a^ (±0.51)	3.01 ^b^ (±0.58)	521.44 ^A^ (±86.37)

Different small letters denote differences in pre- and post- microCT images and after fracture resistance test (α = 0.05). Capital letters compare the groups after fracture resistance test (One-way NOVA and Tukey test). The significance level was set at 5%

**Fig. 2:**
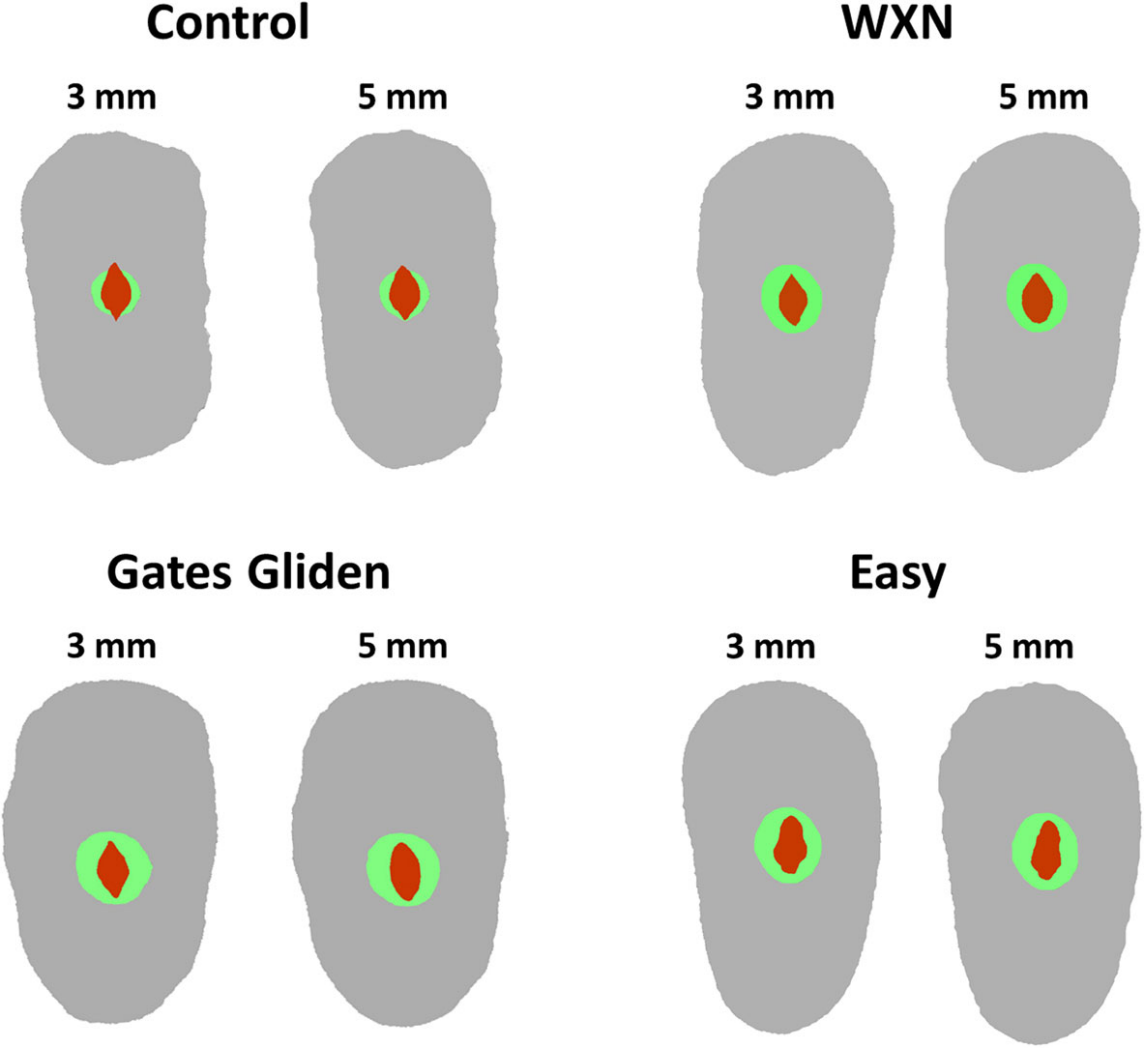
Illustrative micro-CT scans of preparations produced by the tested instruments in 3 mm and 5 mm from the cement-enamel junction (red: preoperative canal; green:postoperative canal)

The dentin thickness presented at baseline was similar in all groups at 3 mm and 5 mm, both for the mesial and distal portion in the cervical third (*P* > .05). Root canal preparation reduced the dentin thickness at 3 mm and 5 mm both in the mesial and distal portion of the root canal (*P* < .05).

The canal volume, up to the fifth millimeter below the enamel-cement junction, at baseline was similar in all groups (*P* > .05). Canal preparation with a 25.07 instrument significantly enlarged the canal space up to the fifth millimeter below the enamel-cement junction (*P* < .05) but with no differences among the groups.

The Gates Glidden group presented the lower fracture resistance values (*P* < .05). WXN, Easy, and Control groups showed similar fracture resistance (*P* > .05). Illustrative micro-CT scans after cervical preflaring and root canal preparation are show in Fig. 1 and 2.

## Discussion

The present study aimed to investigate the fracture resistance of endodontically treated teeth after cervical preflaring and root canal preparation and to assess the volume of the root canal and the amount of remaining root dentin before and after cervical preflaring. The results reject the null hypothesis to fracture resistance, indicating differences among preflaring instruments, and accepting the null hypothesis for the volume of the root canals and amount of remaining dentin.

Traditional endodontics therapy makes the controlled removal of tooth structure beyond getting access to the root canal to facilitate cleaning, shaping, root canal filling and to prevent procedural complications [12]. The access of pulp chamber, along the chamber walls, and around canal orifices, may weaken the resistance of the tooth to fracture under functional loads [13]. Micro-CT scanning was used for sample selection, dentin thickness evaluation, and cervical canal volume. This non-invasive method is considered gold-standard for these outcomes once it permits analyses before and after treatment, reconstruction in two and three dimensions and high level of accuracy and detailing [14]. The micro-CT is a nondestructive model wich allow evaluate the presence of dentin defects before and after root preparation without confounding factors, because each specimen acts as its own control [15].

This study used mandibular incisors because they present mesiodistal flattening which may lead to thin dentin layers after cervical preflaring and canal preparation. And as a consequence, lower fracture resistance values would be obtained. Three instruments were used for cervical preflaring: Gates Glidden, WXN 25.07 and Easy 25.08. The use of Gates-Glidden burs sizes #2 and #3 with a 0.7 mm and 0.9 mm diameter, respectively, was determined based on previous studies that have also used these diameters to promote cervical preparation [16, 17]. WXN (25.07) and Easy (25.08) NiTi instruments were used in continuous rotary motion to prepare the cervical third of the root canals. WXN is one of the files from a six-instrument blister which has a triangular section and an inactive tip. This system provides constant taper throughout the sequence of instruments used. Easy Pro Design S instruments present triple helix and thermal treatment to optimize their mechanical properties and also to promote controlled-memory [1].

Mandibular incisor presents a percentage of long oval canal of 56% [18]. To this cross section a widen circular preparation could weaken or perforate the roots. The lower mean dentin thickness observed after cervical preflaring was 1.02 mm and 1.06 mm in the 5 mm below the cement-enamel junction of the mesial and distal portion respectively. Keeping a minimal residual dentin thickness of more than 1 mm below the cemento-enamel junction as recommended by Pilo [19].

After cervical preflaring and root canal preparation, there was no difference in canal volume and in remaining mesial and distal root dentin between the groups (*P* > .05). Thus, the first null hypothesis was confirmed regarding dentin thickness and volume of root canal. These results occurred due to the dimensions of instruments/bur used in each group, which promoted similar wear in the inner part of the canal.

Root canal anatomy and instrument features must be taken into account when cervical preflaring will be performed [17]. The Gates Glidden #2 did not significantly decrease the residual dentin thickness in the coronal third of mandibular molars, preserving their mechanical integrity [20]. Recently, Flores et al. [16] concluded that Gates Glidden #3 provided adequate cervical preparation of the root canals. All these findings are in accordance with other studies which also evaluated remaining dentin thickness/walls after cervical preflaring [17].

However, none of these studies evaluated the impact of cervical preflaring or the instrument used for this purpose on the fracture of resistance of endodontically treated teeth. The present study assessed this outcome and rejected the null hypothesis. The fracture resistance of teeth submitted to cervical preflaring using Gates Glidden #2 and #3 was lower than those prepared WXN 25.07, Easy 25.08 or with no cervical preparation (control group) (*P* < .05). Instead, the tapered and continuous format of NiTi instruments, Gates Glidden burs have an hourglass format which may explain these unfavorable results because they did not enable uniform load distribution along the root canal walls. The emergence of heat-treated instruments and reciprocating movement, the endodontic literature has demonstrated the absence of the need cervical preflaring [21].

A limitation of this study is unknow the donor age. The strength of radicular dentin could be affected by age factor [22], although this degradation does not exceed the decrease strength of radicular dentin caused by endodontic treatment. More finite element analysis (FEA) studies must be performed to confirm the relationship between the hourglass format promoted by Gates Gliden burs and the stress distribution along the canal walls of preflared teeth. Until now, FEA studies indicate that the access cavity preparation had the greatest influence on tooth strength whilst canal enlargement did not contribute to this process substantially [20, 23].

## Conclusion

Based on the results, it can be concluded that:

1. In the resistance test, the Gates Gliden burs weakened mandibular incisors (*P* < .05) compared to NiTi instruments (*P* > .05).
2. All instruments promoted similar reduction of dentin thickness and increasing in the canal volume at 5 mm and 3 mm below enamel-cement junction.

## Data Availability

All data generated or analysed during this study are included in this published article.

## Abbreviations

NaOCl: sodium hypochlorite
WL: Working length
